# The impact of rifaximin on inflammation and metabolism in alcoholic hepatitis: A randomized clinical trial

**DOI:** 10.1371/journal.pone.0264278

**Published:** 2022-03-14

**Authors:** Nina Kimer, Mads Meldgaard, Ole Hamberg, Thit Mynster Kronborg, Allan M. Lund, Holger Jon Møller, Flemming Bendtsen, Henriette Ytting

**Affiliations:** 1 Gastro Unit, Medical Division, Amager-Hvidovre University Hospital, Hvidovre, Denmark; 2 Department of Internal Medicine, Zealand University Hospital, Koege, Denmark; 3 Department of Hepatology and Gastroenterology, Rigshospitalet, Copenhagen, Denmark; 4 Center for Inherited Metabolic Diseases, Departments of Clinical Genetics and Pediatrics, Rigshospitalet, Copenhagen, Denmark; 5 Department of Clinical Biochemistry, Aarhus University Hospital, Aarhus, Denmark; Kaohsiung Medical University, TAIWAN

## Abstract

**Background and aims:**

Alcoholic hepatitis (AH) is characterized by acute liver failure, neurocognitive impairment and renal failure. Severe inflammatory reactions are also known to occur in AH. Inflammation and bacterial translocation in the gut are thought to have major impact on disease development and progression. The mortality rate for AH is close to 50%. We aimed to assess the efficacy of rifaximin in treating AH and its impact on inflammation and metabolism.

**Methods:**

The trial was approved by relevant authorities (EudraCT no: 2014-02264-33, Scientific Ethics Committee, jr. no: H-1-2014-056). Primary outcomes were changes in metabolic and inflammatory markers. Secondary outcomes were portal hypertension, kidney and neurocognitive function.

**Results:**

Thirty-two patients were randomized to standard medical therapy (SMT) or SMT plus rifaximin, allocation was concealed. Four patients in the SMT group and five patients in the SMT + rifaximin group died due to AH and liver failure. No adverse events related to the study medication were observed. We found no significant differences in amino acids or inflammation markers (IL-2, IL-6, IL-8, IL-10, TNF-α, interferon-γ) between the groups after 28 and 90 days.

**Conclusion:**

Rifaximin does not alter inflammation or metabolism in patients with AH.

## Introduction

Alcoholic hepatitis (AH) develops in response to a considerable intake of alcohol among people with a susceptibility to the disease. The condition is characterized by acute liver failure and presents as jaundice, impaired liver function, neurocognitive impairment and, often, renal failure. Severe inflammatory reactions can be observed histologically, as well as biochemically with increased levels of inflammatory mediators in the blood [1, 2].

The prevalence of AH is increasing and has a 50% mortality rate within eight weeks of onset [3, 4]. AH is seen in patients with verified alcoholic liver cirrhosis who are actively abusing alcohol, but also among people with alcohol dependency or excessive alcohol intake without signs of cirrhosis, and occurs as both acute liver failure and acute-on-chronic liver failure [5, 6].

Bacterial translocation has been hypothesized as a key pathogenic driver of systemic inflammation in acute-on-chronic liver failure [7, 8]. In the presence of chronic liver disease, an increased permeability in the gut membrane causes translocation of bacteria and bacterial pathogens into the lymphatic and blood systems, activating inflammatory mediators in the liver [9]. This may be further aggravated by alcohol consumption, which in the intestinal mucosa leads to destruction of the epithelial tight junctions, increasing gut permeability and raising levels of endotoxins in the blood stream [10, 11].

Increased levels of endotoxins are precursors of hepatic and systemic inflammation and are also involved in the chronic inflammation seen in alcoholic liver disease [12]. Endotoxins initiate an inflammatory response by binding to macrophages and monocytes, among others [13]. The liver comprises the largest reserve of macrophages in the human body, and after activation by endotoxins they release pro-inflammatory and anti-inflammatory cytokines, such as Tumor necrosis factor-alpha (TNF-α), and interleukins 1, 2, 6, 8 and 10. In patients with cirrhosis the inflammatory response is more potent and the clearance of endotoxins depressed [14, 15].

The systemic inflammation observed in AH may be a result of the pro-inflammatory effects of both alcohol and liver disease on bacterial translocation. Excessive alcohol intake (causing increased gut permeability and a greater impact of pro-inflammatory markers such as NF-kB and free radicals), and impaired liver function together with impaired macrophage activation all contribute to an uncontrolled inflammatory response to the bacterial translocation [16, 17]. It is therefore hypothesized that bacterial translocation plays an important role in driving the systemic inflammation in AH [18].

Rifaximin is a non-absorbable antibiotic with a broad-spectrum effect on both gram positive and gram negative bacteria, and is effective in preventing recurrent hepatic encephalopathy [19].

Rifaximin may modulate the bacterial composition in the gut towards bacteria with lower production of ammonia and toxins, thus reducing the quantities of circulating endotoxins and cytokines [20, 21].

In a randomized, controlled trial we investigated the effect of rifaximin on inflammatory and metabolic mediators in patients with AH, and assessed its effect on liver function, (including portal hypertension), hepatic encephalopathy and kidney function.

## Participants and methods

The study was an open-label, randomized, controlled trial initiated by the investigators and conducted at a single tertiary center, the Department of Hepatology, Rigshospitalet, Denmark. Patients were enrolled between September 2015 and May 2018. The trial was registered at www.clinicaltrialsregister.eu (EudraCT no: 2014-02264-33, date: 25-05-2014) and the study protocol was approved by the Danish Medicines Agency (date: 25-08-2014) and the European Medicines Agency (EudraCT no: 2014-02264-33). The study protocol conformed to the ethical guidelines of the 1975 Declaration of Helsinki and was approved by the Scientific Ethics Committee of the Capital Region of Denmark (journal no: H-1-2014-056, date: 10-10-2014). The Good Clinical Practice (GCP) Unit of Copenhagen University Hospital served as GCP monitor of the trial.

### Sample size

The study was exploratory in nature. Power calculation was based on a reduction in LPS, an inflammation marker included in the primary endpoints. Using a 2-sided 2-sample test (www.stat.ubc.ca), a sample size of fifteen patients in each group yielded 85% power to detect a 30% reduction in LPS in the control group, at a 5% level of significance.

### Outcome measures

The primary outcome measures were changes in inflammatory and metabolic markers measured on days 28 and 90.

The secondary outcomes were effects on portal pressure (measured by hepatic venous pressure gradient (HVPG)), kidney function, and neurocognitive function (measured by continuous reaction time and number connection tests conducted on days 28 and 90).

### Inclusion and exclusion criteria

Inclusion criteria were: i) alcohol intake of more than three units of alcohol (40 grams) per day for more than three months or more than 10 units per day for more than one month; ii) and rapid development of jaundice (in less than 14 days) and plasma bilirubin above 50 μmol/liter; iii) and elimination of mechanic or obstructive causes of cholestasis by CT scan or ultrasound. Exclusion criteria were pregnancy, being younger than 18 years, incapability of giving informed consent, treatment with rifaximin within the last four weeks, malignant disease, intolerance or allergy towards rifaximin or tablet contents, or intestinal obstruction contraindicating treatment with rifaximin.

### Study design

Patients were randomized 1:1 to either arm of the study, based on a list of random numbers generated by an independent physician, MD Gro Askgaard, not otherwise involved in the study. The trial was open-label, but allocation was concealed using serially numbered, opaque, sealed envelopes. Stratification according to the Glasgow Alcoholic Hepatitis Score (GAHS) was used to ensure equal distribution of patients according to severity in each group. All patients gave written consent for participation based on written and oral information given to them prior to inclusion.

Patients were randomized to standard medical therapy (SMT), or SMT + rifaximin (Xifaxan^TM^) 550 mg three times daily for four weeks.

SMT included pentoxifylline 400 mg x 3 daily for 2–4 weeks. In addition, patients were given nutrition, fluid therapy, systemic antibiotics and possibly Vitamin K, if suggested by the treating physician. In case of improvement), prednisolone 40 mg per day was administered.

Rifaximin was administered by a ward nurse at eight-hour intervals. The nurse observed and documented oral intake of the tablet. If the patient was incapable of oral intake, the tablets were crushed and administered via a gastric tube. Patients with an intake of at least 80% of the study medication were considered compliant. Rifaximin was prescribed and distributed as standard medication from the hospital pharmacy, with additional labelling according to Good Medical Practice Annex 13 [22]. ID number, name of participant and name of investigator were provided on each container, and in the event the participant was discharged before completing four weeks of treatment, the container was given to them to continue treatment at home. The study protocol is available in the Supplementary Materials.

### Methods of investigation

Clinical history, demographic data and standard biochemistry were all collected. On the day of inclusion, patients were assessed for their psychometric hepatic encephalopathy score (PHES) [23], and clinical scores for AH, Maddrey’s discriminant function for AH, the Age-Bilirubin-INR-Creatinine (ABIC) score, and GAHS score [3, 24, 25].

Liver vein catheterization was performed before day 7, with access via the right antebrachial vein, as described elsewhere [26]. HVPG was measured as the difference between the free hepatic venous pressure and the wedged hepatic venous pressure, and as a mean of measurements from three different positions. Liver stiffness was measured by FibroScan before day 7.

Biochemical and clinical assessments were repeated weekly for the first four weeks. Participants were treated during hospital admission, and in case of discharge before day 28, outpatient visits were scheduled for days 28 and 90. Samples were frozen at -80 degrees Celsius to be used for later measurement of inflammatory markers.

The investigative procedure is illustrated in S1 Fig. Patients’ 90-day follow-up was retrieved from the Danish Central Person Registry and their medical health records.

The inflammation markers–interleukins (IL-2, IL-6, IL-8, IL-10), TNF-α and interferon-γ (INF-γ)–were analyzed with a commercially available, high-sensitivity V-PLEX Proinflammatory Panel 1 Human Kit (cat. no. K15049D from Meso Scale Discovery).

LBP was measured with a commercially available kit (Hycult Biotech (Nordic Biosite), cat. no. HK315-02). Soluble sCD163 was determined using an in-house sandwich enzyme-linked immunosorbent assay (ELISA), using a BEP-2000 (Dade Behring), as described elsewhere, and using the same reference intervals for healthy individuals (0.69 to 3.86 mg/l) [27].

The amino acids glutamine, leucine, isoleucine, valine, phenylalanine, tyrosine, tryptophane were analyzed by MassTrak^TM^ and Amino Acid Solution Kit^TM^, both of which are commercially available.

Blood samples were placed on ice immediately, centrifuged at 4 degrees Celsius, and plasma was isolated and frozen at -80 degrees Celsius. For the amino acids, sulfosalisylic acid and 6-aminoquinolyl-N-hydroxysuccinimidyl carbamate were added to the plasma and analyses performed using UV ultra-high performance chromatography.

### Statistical analysis

All data were analyzed using the statistical software GraphPad Prism9.0 (San Diego, CA 92108). For both primary and secondary outcomes, groups were compared using non-parametric Mann–Whitney *t*-test of the mean differences between values at baseline and on day 28, assuming a non-Gaussian distribution of data.

We planned repeated measures analysis of variance with multiple comparisons between baseline, day 28, and day 90, but due to considerable attrition during the study we discarded the plan and instead conducted a subgroup analysis between baseline and day 90 measurements using non-parametric Mann–Whitney t-test of mean differences with the remaining participants.

## Results

**Fig 1:**
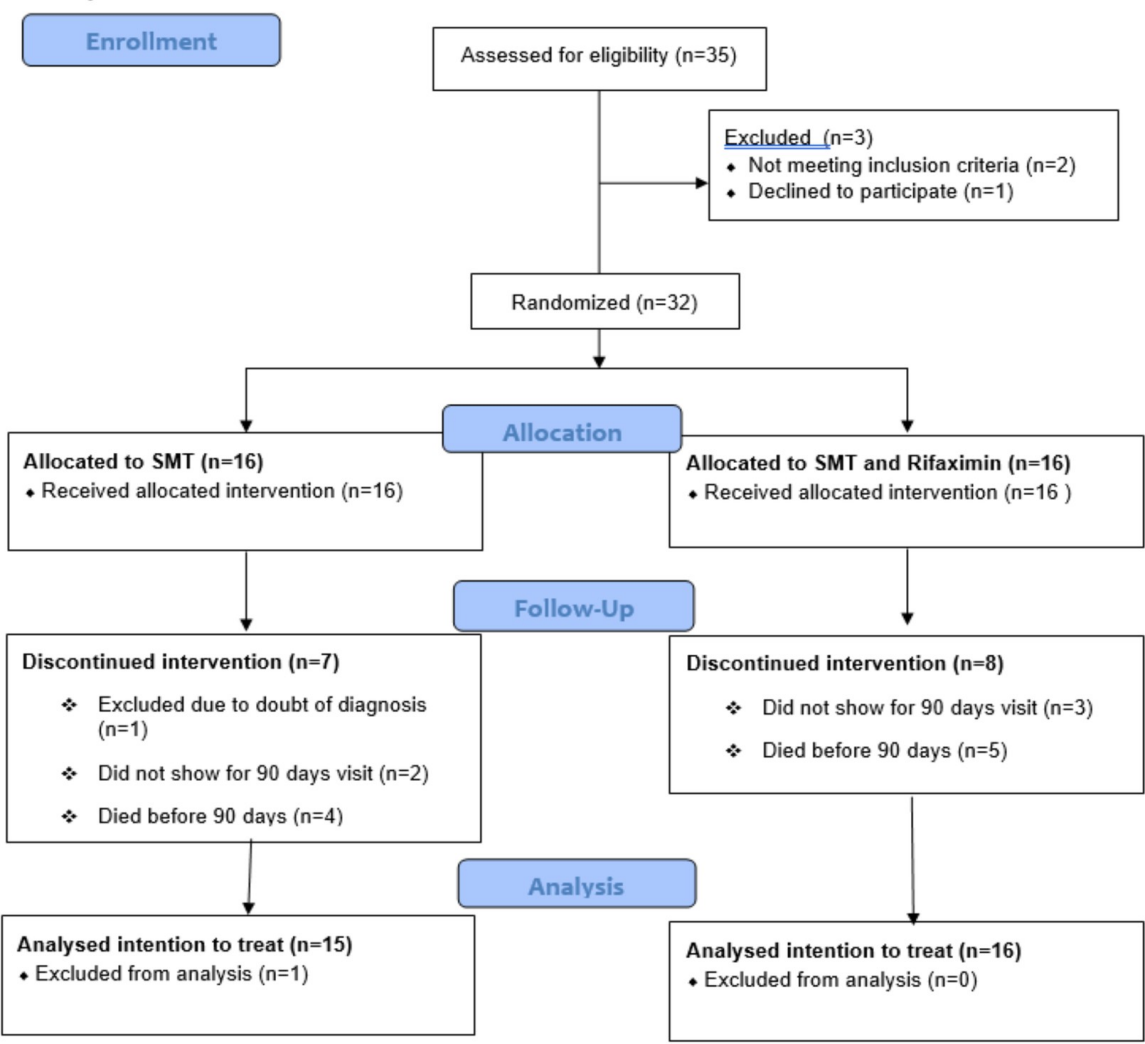
CONSORT flowchart.

Between September 2015 and May 2018, 35 patients were screened for eligibility and a total of 32 patients were randomized to SMT or SMT + rifaximin (Fig 1). One patient allocated to SMT was excluded after ten days of treatment due to reasonable doubt as to their diagnosis and was also excluded from the data analysis. Of the remaining 31 participants, 21 patients were male, and the median age was 51 years (Table 1, patient characteristics). Twenty-two participants had ascites at baseline, 12 in the SMT group, 10 in the SMT + rifaximin group. Nineteen participants had an abnormal PHES score indicating minimal HE at baseline, 11 in the SMT group, nine in the SMT + rifaximin group.

**Table 1:** Baseline characteristics.

	Total N = 31	Standard treatment N = 15	Standard treatment + rifaximin	*P*-value
			N = 16	
Age	51 (33–74)	52 (33–67)	51.5 (35–74)	0.590
Male	21 (67.7%)	8 (53.3%)	13 (81.3%)	NA
Time from admission to inclusion, days	11.5 (0–43)	10 (0–27)	12 (2–43)	NA
Encephalopathy, PHES	-9 (-1 to -18)	-8 (-1 to -16)	-12.5 (-4 to -18)	0.883
SIRS upon admission	5 (15.6%)	2 (12.5%)	3 (18.8%)	NA
Hepatic venous pressure gradient, mmHg	18 (7–26)	18 (7–26)	18 (8–21.5)	0.677
FibroScan kPa	72 (45–75)	69 (45–75)	75 (48–75)	0.141
Laboratory tests				
*Bilirubin*, *μmol/l*	343 (51–789)	297 (56–789)	370 (51–497)	0.091
*Albumin*, *g/l*	20 (14–36)	20.0 (14–36)	20.5 (14–35)	0.822
*ALAT*, *U/l*	44 (20–326)	44 (25–129)	43 (20–326)	0.961
*Creatinine*, *ml/min*	72 (27–196)	67 (44–196)	81.5 (27–141)	0.633
*White cell count*, *e9/l*	15.7 (3.6–35.2)	17.2 (4.8–35.2)	12.55 (3.6–24.4)	0.172
*C-reactive protein*, *mg/l*	29 (2–64)	29 (4–61)	29.5 (2–64)	0.868
*Platelets*, *e9/l*	183 (29–431)	190 (53–431)	177 (29–325)	0.379
*Coag*. *factor II*,*VII*,*X*: *pp*	0.23 (0.1–0.48)	0.29 (0.13–0.48)	0.23 (0.1–0.37)	0.069
*Ammonia*, μmol/l*	66.5 (24–254)	75 (25–147)	56 (27–254)	0.394
MELD	27.4 (18.5–34.6)	24.9 (18.5–34.6)	28.85 (19.6–33.1)	0.185
GAHS	10 (8–12)	10 (8–12)	10 (8–11)	0.520
Maddrey	79.4 (24.2–172.5)	60.1 (50.3–138.3)	97.8 (24.2–172.5)	0.049
ABIC	8.89 (6.5–11.7)	8.62 (6.5–11.7)	9.25 (7.84–11.21)	0.100

Data are given as a median and range. ALAT: Alanine transaminase; *Ammonia was measured by arterial blood.

SIRS: Systemic inflammatory response syndrome; PHES: Psychometric hepatic encephalopathy score; MELD: Model for end-stage liver disease; GAHS: Glasgow alcoholic hepatitis score; ABIC: Composite score of Age, Bilirubin, INR and Creatinine.

Four patients in the SMT group and five patients in the SMT + rifaximin group died before day 90, and in all cases the cause of death was AH and liver failure. Five patients did not show up for their final visit on day 90 (Table 2). We observed no adverse events related to the study medication.

**Table 2:** Attrition.

	Standard treatment	Standard treatment + rifaximin
Mortality	4	5
Due to HRS	2	3
Due to AH and HE	1	1
Due to Sepsis and liver failure	1	1
Development of HRS	2	4
Attrition before end of study	2 (no-show for visit at day 90)	3 (no-show for visit at day 90)
Adverse events possibly related to study medication	0	0

HRS: Hepato-renal syndrome; HE: Hepatic encephalopathy.

Inflammatory markers were elevated at baseline and after 28 days in all patients and compared to reference values from healthy individuals [28]. IL-6 and IL8 were elevated at least 100-fold in study participants as compared to reference values; IL-10, TNF-α and INF-γ were elevated two- to five-fold (Table 3). These levels decreased over time, but the changes were not significant and we did not find any significant effect of rifaximin different to that of SMT after 28 days of treatment. Levels of the amino acid glutamine decreased after 28 days in the SMT group, but increased in the rifaximin group, where the mean difference in the SMT group was 244.5 μmol/l (SD 666.5) versus -43.73 μmol/l (SD 121.3) in the SMT + rifaximin group (*p* = 0.029) (Table 3). We did not detect any other significant differences in the abundance of amino acids between the SMT and rifaximin groups after 28 days.

**Table 3:** Primary outcomes of inflammation and metabolism.

**Inflammatory markers**							
	**Standard treatment N = 15**			**Standard treatment + Rifaximin N = 16**			***P*-value**
	**Baseline**	**28 days**	**Mean diff**	**Baseline**	**28 days**	**Mean diff**	
Procalcitonin μg/l	0.82 (0.8757)	0.5347 (0.4571)	0.3714 (0.687)	0.6107 (0.4590)	0.5563 (0.4372)	0.0207 (0.5326)	0.229
sCD163 mg/l	16.97 (5.22)	15.72 (10.84)	1.413 (8.775)	17.32 (6.447)	17.43 (10.84)	-0.4438 (7.532)	0.340
Lipid-binding protein μg/l	21.53 (13.75)	17.78 (7.644)	3.631 (6.591)	18.97 (10.39)	15.92 (8.570)	3.048 (5.028)	0.877
Interferon-γ pg/ml	6.34 (10.69)	25.59 (46.38)	-19.25 (48.06)	4.815 (5.881)	9.00 (8.055)	-4.823 (8.55)	0.363
Interleukin 10 pg/ml	2.01 (1.35)	2.033 (1.883)	-0.02 (20.33)	1.650 (1.120)	1.481 (1.188)	0.1625 (1.298)	0.899
Interleukin 2 pg/ml	0.2100 (0.1663)	0.1125 (0.0991)	0.09 (0.1792)	0.1872 (0.0641)	0.0818 (0.07508)	0.0375 (0.09161)	0.757
Interleukin 6 pg/ml	12.27 (10.58)	11.39 (10.63)	0.88 (15.24)	15.32 (10.11)	16.69 (18.88)	-1.375 (20.95)	0.953
Interleukin 8 pg/ml	360.2 (373.9)	118.4 (116.9)	241.8 (340.6)	376.8 (456.7)	194.1 (251.7)	191.3 (322.4)	0.595
TNF-α pg/ml	5.87 (2.49)	5.953 (3.647)	-0.080 (3.849)	6.500 (5.146)	5.638 (1.728)	0.92 (4.998)	0.616
**Amino acids**							
	**Standard treatment**			**Standard treatment + rifaximin**			
	**Baseline**	**28 days**	**Mean diff**	**Baseline**	**28 days**	**Mean diff**	***P*-value**
Glutamine μmol/l	735.2 (711.3)	484.8 (113.1)	244.5 (666.5)	582.8 (191.1)	617.5 (207.1)	-43.73 (121.3)	**0.0295**
Tyrosine μmol/l	114.1 (68.4)	94.20 (31.17)	20.53 (60.28)	146.1 (76.24)	156.6 (95.19)	-10.00 (49.92)	0.127
Valine μmol/l	136.5 (35.3)	118.6 (57.10)	17.87 (69.46)	147.6 (49.54)	142.1 (49.43)	5.438 (52.56)	0.552
Isoleucine μmol/l	41.27 (13.60)	37.13 (16.31)	4.133 (23.02)	44.81 (15.07)	43.44 (14.90)	0.75 (15.41)	0.451
Leucine μmol/l	75.07 (18.75)	65.27 (31.76)	9.8 (34.68)	83.69 (23.97)	80.94 (30.78)	2.75 (32.03)	0.452
Fenylalanine μmol/l	78.13 (32.21)	76.47 (44.21)	1.00 (52.27)	106.9 (90.06)	95.38 (49.01)	11.56 (63.67)	0.565
Tryptofane μmol/l	45.67 (31.00)	38.60 (19.01)	7.20 (0.0267)	61.38 (60.28)	55.50 (32.20)	6.4 (67.76)	0.602

Data are given as a mean and SD.

Mean diff; Mean difference between baseline and 28 days.

Eight patients in the SMT group and seven patients in the SMT + rifaximin group had comparable measures of inflammation markers after 90 days. The remaining nine patients in the SMT group and eight patients in the SMT + rifaximin group had amino acids analysed after 90 days. No significant differences between the levels of amino acids at baseline and after 90 days were found in either group (S1 Table).

The secondary outcomes of liver function and kidney function (Creatinine and Urea) were also found to be unaffected by rifaximin (Table 4).

**Table 4:** Secondary outcomes of liver function.

	Standard treatment			Standard treatment + rifaximin			
	Baseline	28 days	Mean diff	Baseline	28 days	Mean diff	*P-*value
Creatinine μmol/l	85.80 (46.71)	79.80 (32.35)	6.00 (53.44)	80.19 (30.45)	106.4 (67.26)	-30.63 (75.25)	0.148
Urea μmol/l	7.287 (5.06)	7.607 (4.927)	-0.32 (6.625)	7.069 (4.938)	11.37 (10.89)	-4.238 (9.965)	0.565
eGFR ml/min	74.30 (30.62)	67.87 (31.59)	8.00 (39.35)	88.13 (47.38)	77.63 (37.28)	9.133 (26.55)	0.633
Ammonia μmol/l	75.5 (32.33)	61.73 (23.63)	14.43 (38.50)	75.06 (53.77)	73.25 (23.30)	1.813 (56.46)	0.051
PHES	-8.833 (4.629)	-6.75 (4.475)	1.50 (4.475)	-11.40 (5.168)	-9.5 (6.519)	1.900 (5.99)	0.684
HVPG ^*^ mmHg	15.33 (6.38)	12.55 (6.02)	4.33 (5.29)	16.67 (4.25)	13.73 (4.125)	3.667 (3.832)	0.990
FibroScan kPa	65.82 (10.77)	50.52 (19.53)	12.75 (21.35)	68.47 (9.72)	65.89 (13.26)	2.57 (15.72)	0.430

eGFR: estimated glomerular filtration rate. Mean diff: Mean difference between baseline and 28 days. *15 patients (nine in the SMT + rifaximin group and seven in the SMT group) had a repeat HVPG measurement taken on day 90. Eleven patients in the SMT group and ten patients in the SMT + rifaximin group had a repeat FibroScan on day 90.

During the study, four patients in the SMT group received pentoxifylline (400 mg three times daily for four weeks), and nine patients received prednisolone (initial 40–80 mg with phasing out regimes) as standard medical treatment. In the SMT + rifaximin group five patients received pentoxifylline, and nine patients received prednisolone. SMT treatment with either prednisolone or pentoxifylline was evenly distributed between the two groups. Switching between standard therapies was based on patients’ Lille scores (S2 Table).

## Discussion

In a randomized trial we investigated the effetcs of rifaximin on inflammation and metabolism when adding it to standard medical treatment for patients with severe AH. These exploratory outcomes, as well as liver and kidney function, were found to be unaffected by rifaximin treatment in AH.

Standard medical therapy included both pentoxifylline and prednisolone, and was prescribed at the discretion of the treating consultant. Therefore, some patients received both treatments, while others started prednisolone only later in the trial. At the time the study was conducted, prednisolone and pentoxifylline were considered equally efficacious [29], however later guidelines have questioned the efficacy of pentoxifylline and favored prednisolone [30]. The study was conducted at a tertiary centre and the dosages of treatment initiated at admission and prior to inclusion was not always registered in detail. It is unclear whether the variations in standard therapy impacted inflammation markers and metabolism in AH, but due to the trial’s randomized allocation of patients prednisolone and pentoxifylline treatment was evenly distributed between the two groups (S2 Table).

Mortality was high, but similar to the rates reported in prior studies of AH [4].

Based on previous findings regarding the effects of rifaximin on portal hypertensive complications, and the presumed effect on circulating endotoxin levels, we had expected a decrease in the inflammatory parameters in patients treated with rifaximin [31–33]. This was despite a previous study in stable decompensated cirrhosis not finding a significant impact of rifaximin on inflammation and bacterial translocation [34, 35]. The levels of inflammatory markers were found to be higher in our study, which might reflect that AH has a greater impact on inflammation and metabolism than cirrhosis alone.

The levels of amino acids were generally within normal reference ranges, although tyrosine and phenylalanine were slightly increased (as seen in liver insufficiency), and branched chain amino acids were slightly decreased, probably reflecting liver disease and decreased protein intake. We observed no consistent changes or differences between the two groups during the study. With regards to glutamine a significant increase of plasma concentration was seen in rifaximin-treated patients during the 28-day treatment period, whereas a small decline was observed in the SMT group. If this is an effect of rifaximin it might be due to a modulation of the metabolic stress seen in severe AH that leads to glutamine deficiency. A possible mechanism for this could be rifaximin-mediated upregulation of the hepatic glutamine synthetase, which could also explain the effect of rifaximin on hepatic encephalopathy, as glutamine synthetase serves as scavenger for ammonia [36]. Alternatively, it could be that rifaximin increases the intestinal production of glutamine [37]. For all other amino acids measured in our study there was a minor, non-significant decline of plasma concentrations during the treatment period in both groups. The significant difference in glutamine from baseline to day 28 (but not from baseline to day 90) could be the result of a random decrease in the SMT group and an increase in the SMT + rifaximin group, especially as no changes were observed in concentrations of ammonia.

We observed an increase in the inflammation markers at baseline, especially IL-10. Prior studies have demonstrated increased levels of IL-10 and IL-10 producing T-cells in AH compared to both patients with cirrhosis and healthy controls [38, 39]. A prior study have also demonstrated an overrepresentation of polymorphisms in the genes encoding for cytokines patients with alcohol-related liver damage [40]. We did not find an effect of rifaximin on inflammation markers, which is in consent with prior studies in alcohol related liver disease [35]. The clinical impact of increased inflammation markers associated with AH is unclear and further studies are needed to assess the clinical application of inflammation markers in diagnosis and monitoring of AH.

The present study has several weaknesses. Due to its exploratory nature, the sample size is limited and larger, randomized trials investigating AH could throw more light on the alterations in both inflammation and metabolism as they relate to an improvement in disease course. However, we were not able to determine whether these effects were important for recovery from AH. In addition, the mixture of drug treatments given to the patients in this study might have affected our results.

The substantial drop-out over the course of the 90 days, due to both high mortality and low compliance, omits the use of a mixed effects model for multiple comparisons over time. In a model such as this, results can be misleading if the value of the missing parameters is related to the cause of their absence, and in this case it could be that progression or improvement of disease might have influenced the completeness of our data. However, the small sample size and consistency of non-significance across parameters leads us to believe that these results would not have reached statistical significance even with fewer drop-outs.

The study’s strengths are its randomized design and the thorough characterization of a complex disease entity. In addition, the robustness of the non-significant results from comparisons between and within groups leads us to believe that rifaximin does not alter any of the parameters we assessed.

The disease course of AH is acute and progresses rapidly. Inflammation markers and levels of amino acids are normally higher in the early stages, although we could not replicate this in the present study. A spontaneous improvement indicated by a reduction in inflammation markers was observed in both groups, irrespective of treatment. Any beneficial effect of rifaximin could have been disguised by both spontaneous improvement in the condition over time and treatment with prednisolone or pentoxifylline in both groups.

The effects of rifaximin in liver cirrhosis and AH have previously been explored in relation to the impact on portal hypertension, the gut microbiome and inflammation [35, 41, 42]. Although rifaximin may alter the gut microbiome in favor of less pathogenic bacteria and help to alleviate dysbiosis, its effect on bacterial translocation and pathogen-associated molecular patterns that activate the inflammasome in the liver has not been established. Furthermore, the effect of rifaximin might be superseded by the inflammatory cascades activated in AH [7, 21, 43].

Recent research has explored the metabolic mechanisms involved in both progression and remission of AH, suggesting potential diagnostic markers and novel drug targets [44, 45]. Future studies of the inflammation cascades driving AH and the metabolic processes involved could facilitate a better understanding of the disease and ways in which to treat it.

## Conclusion

Rifaximin does not appear to alter inflammation or metabolism in AH and our results do not support indications for use of rifaximin in AH, unless the indication is hepatic encephalopathy. The decrease we observed in inflammation markers over time may be due to spontaneous improvement of the disease, or SMT provided to both groups, or a combination of the two.

## Supporting information

S1 Fig Investigative program. (PNG)

S1 Table Primary outcomes after 90 days. (DOCX)

S2 Table Standard medical treatment. (DOCX)

S1 File RifAH protocol. (DOCX)

S1 Data Data file anonymized. (XLSX)

S1 Checklist CONSORT checklist. (DOC)

## Abbreviations

ABIC: Age-Bilirubin-INR-Creatinine
ALAT: Alanine Transaminase
AH: Alcoholic hepatitis
ELISA: enzyme-linked immunosorbent assay
eGFR: estimated Glomerular filtration rate
GAHS: Glasgow Alcoholic Hepatitis Score
GCP: Good Clinical Practice
HE: Hepatic encephalopathy
HRS: Hepato-renal syndrome
HVPG: hepatic venous pressure gradient
INF-γ: Interferon-γ
IL: Interleukin
MELD: Model for End-stage Liver Disease
PHES: psychometric hepatic encephalopathy score
SD: Standard Deviation
SMT: standard medical therapy
SIRS: Systemic Inflammatory Response Syndrome
TNF-α: Tumor necrosis factor-alpha
