# Supplementary figures and images for: The impact of rifaximin on inflammation and metabolism in alcoholic hepatitis: A randomized clinical trial
Source: PLoS One. 2022 Mar 14;17(3):e0264278. doi: 10.1371/journal.pone.0264278 (PMC8920190; doi:10.1371/journal.pone.0264278)

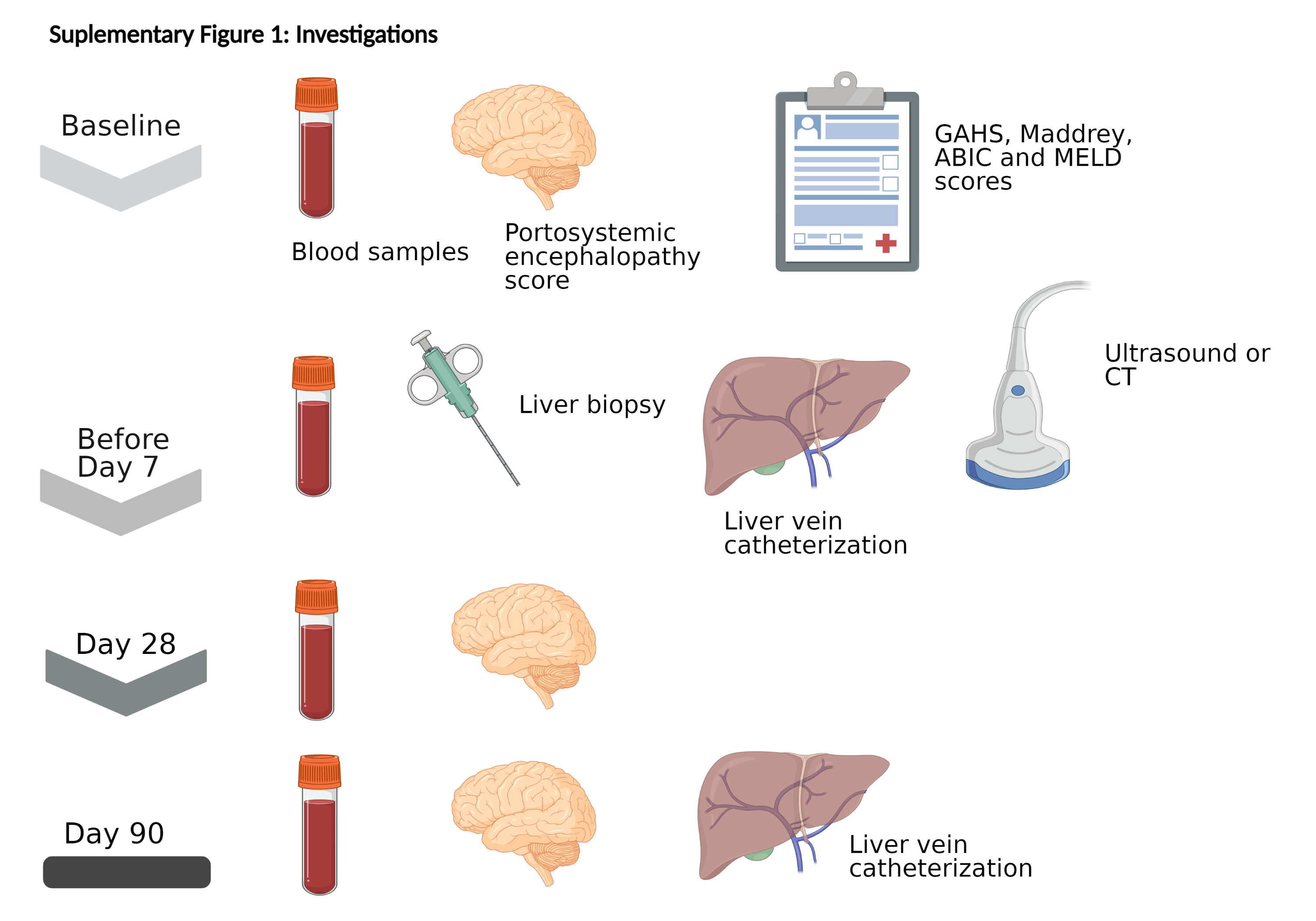

Supplement: S1 Fig — (PNG) [file pone.0264278.s001.png]
