# Supplementary material for: The impact of rifaximin on inflammation and metabolism in alcoholic hepatitis: A randomized clinical trial
Source: PLoS One. 2022 Mar 14;17(3):e0264278. doi: 10.1371/journal.pone.0264278 (PMC8920190; doi:10.1371/journal.pone.0264278)
Supplement: S1 Table — (DOCX) [file pone.0264278.s002.docx]

**Supplementary Table 1: Primary outcomes after 90 days**

| **Inflammatory markers** | | | | | | | |
| --- | --- | --- | --- | --- | --- | --- | --- |
|  | **Standard treatment**  **N=8** | | | **Standard treatment + Rifaximin**  **N=7** | | | ***P*-value** |
|  | **Baseline** | **90 days** | **Mean diff** | **Baseline** | **90 days** | **Mean diff** |  |
| Interferon-γ pg/ml | 7.84 (14.1) | 3.50 (2.05) | 4.34 (12,6) | 4.62 (5.50) | 4.08 (2.52) | 0.533 (4.70) | 0.687 |
| Interleukin 10. pg/ml | 1.99 (0.863) | 0.463 (0.262) | 1,74 (0.776) | 1.66 (1.01) | 0.543 (0.355) | 1.11 (0.828) | 0.101 |
| Interleukin 6 pg/ml | 16.6 (12.7) | 2.39 (1.53) | 14.3 (12.7) | 17.40 (11.40) | 4.93 (3.76) | 12.80 (8.69) | 0.955 |
| Interleukin 8 pg/ml | 427,0 (418,0) | 26.9 (25.6) | 400.0 (402.0) | 355.0 (379.0) | 36.6 (30.3) | 318.0 (359.0) | 0.779 |
| TNF-α. pg/ml | 6,03 (2.30) | 3.93 (1.79) | 2.10 (2.50) | 8.91 (6.96) | 4.39 (2.13) | 4.53 (6.79) | 0.802 |
| **Amino acids** | | | | | | | |
|  | **Standard treatment**  **N=9** | | | **Standard treatment + rifaximin**  **N=8** | | |  |
|  | **Baseline** | **90 days** | **Mean diff** | **Baseline** | **90 days** | **Mean diff** | ***P*-value** |
| Glutamine µmol/l | 496.0 (103.0) | 641.0 (112.0) | -145.0 (180.0) | 509.0 (162.0) | 615.0 (66.10) | -108.0 (115.0) | 0.835 |
| Tyrosine µmol/l | 107.0 (44.0) | 111.0 (54.5) | -3.67 (45.6) | 114.0 (45.7) | 112.0 (42.2) | 1.13 (16.70) | 0.333 |
| Valine  µmol/l | 129.0 (29.2) | 133.0 (30.0) | -3.89 (39.6) | 134.0 (24.40) | 135.0 (41.7) | -1.25 (38.10) | 0.725 |
| Isoleucine µmol/l | 39.3 (8.19) | 39.8 (9.19) | -0.222 (11.7) | 42.5 (14.80) | 41.0 (12.0) | 1.50 (14.40) | 0.795 |
| Leucine µmol/l | 67.60 (15.10) | 70.0 (19.20) | -7.11 (20.80) | 80.0 (19.3) | 73.30  (23.70) | 6.75 (21.20) | 0.146 |
| Fenylalanine µmol/l | 68.60 (16.0) | 61.0 (17.80) | 7.56 (16.4) | 80.3 (22.90) | 67.0 (18.3) | 13.30 (13.30) | 0.437 |
| Tryptofane µmol/l | 41.80 (21.20) | 42.80 (9.87) | -1.0 (24.2) | 46.30 (16.10) | 42.0 (14.60) | 4.25 (18.20) | 0.525 |

*Data are given as a mean and SD. Procalcitonin, LBP and sCD163 were not measured after 90 days. IL2 was undetectable after 90 days in a total of 27 patients (13 in the SMT group, 14 in the SMT + rifaximin group)*
