# Supplementary material for: The impact of rifaximin on inflammation and metabolism in alcoholic hepatitis: A randomized clinical trial
Source: PLoS One. 2022 Mar 14;17(3):e0264278. doi: 10.1371/journal.pone.0264278 (PMC8920190; doi:10.1371/journal.pone.0264278)
Supplement: S2 Table — (DOCX) [file pone.0264278.s003.docx]

**Supplementary Table 2: Standard medical treatment**

| **Treatment** | **Standard Treatment** | **Standard treatment + rifaximin** |
| --- | --- | --- |
| Pentoxifylline 400 mg x 3 daily, minimum 28 days | 3 |  |
| Pentoxifylline 400 mg x 3 daily, max 15 days, switch to prednisolone 40 mg per day  *Considered improvement* |  | 4 |
| Pentoxifylline 400 mg x 3 daily, max 15 days  *Considered improvement* | 1 | 1 |
| Prednisolone 40-80 mg per day, min 28 days  *Considered responders* | 5 | 4 |
| Prednisolone 40-80 mg per day, max 15 days  *Considered non-responders* | 4 | 5 |
| Symptomatic  *IV fluids and AB, but no prednisolone or pentoxifylline* | 2 | 2 |
