# Supplementary material for: The impact of rifaximin on inflammation and metabolism in alcoholic hepatitis: A randomized clinical trial
Source: PLoS One. 2022 Mar 14;17(3):e0264278. doi: 10.1371/journal.pone.0264278 (PMC8920190; doi:10.1371/journal.pone.0264278)
Supplement: S1 File — (DOCX) [file pone.0264278.s004.docx]

**Rifaximin in alcoholic hepatitis:**

**Effect on inflammatory and metabolic markers**

**Project group**

Primary investigator /

Chief Physician Ph.D. Henriette Ytting

Department of Hepatology A 2121, Rigshospitalet

Blegdamsvej 9, 2100 Copenhagen Ø

Date Signature:

Sponsor and clinical manager :

Head of clinic, Chief Physician, Med.Sc.D, Ole Hamberg

Department of Hepatology A 2121, Rigshospitalet

Blegdamsvej 9, 2100 Copenhagen Ø

Date Signature:

Other investigators :

Chief Physician Ph.D. Mette Kjær

Senior house officer, MD Johan Rohde

The experiment will be performed in accordance with the present trial protocol, ICH-GCP guideline and applicable regulatory requirements / legislation. Standard procedures for quality control and quality assurance will be followed.

The monitoring of the study in accordance with GCP is handled by the GCP unit at Copenhagen University Hospital.

Partners:

Chief physician, Med.Sc.D, PhD, Ruth Frikke-Schmidt, Dept. of clinical biochemistry, Rigshospitalet.

Chief physician Med.Sc.D. Allan M. Lund, Head, Center for Congenital Metabolic Diseases, Clinical Genetics Clinic, Rigshospitalet.

**The project**

"Rifaximin in alcoholic hepatitis: effect on inflammatory and metabolic markers" is a researcher-initiated, basic research project which is independent of financial support and commercial interest from the pharmaceutical industry. The trial is Phase II-III exploratory, as alcoholic hepatitis is not in itself an approved indication for rifaximin treatment.

**Time schedule**

Expected start: September 1, 2014

Expected completion: September 1, 2017

**Location**

Hepatology Clinic A, Rigshospitalet

**Hypotheses**

1. Treatment with rifaximin in alcoholic hepatitis reduces bacterial translocation and alters the inflammatory and metabolic response. Secondary to this effect, rifaximin modifies complications of alcoholic hepatitis in the form of hepatic impairment, increased portal pressure, affected cerebral function, and decreased renal function

**Primary aim**

To investigate the effect of rifaximin on inflammatory and metabolic mediators in patients with alcoholic hepatitis.

Secondarily, to examine the possible effect on the liver function, portal pressure, cognitive changes due to impaired liver function (hepatic encephalopathy) and renal function .

**Background**

Alcoholic hepatitis (AH) develops in presumably predisposed individuals, in response to significant alcohol intake. The condition is characterized by sudden development of signs of liver failure with jaundice, impaired liver function, disturbances of consciousness and impaired renal function. Histologically, a severe acute inflammatory reaction in the liver is seen and highly elevated proinflammatory mediators are present in the blood.

The condition, which is seen with increasing frequency in Denmark with currently around 400 cases per year, is associated with high mortality of more than 50 % within 8 weeks (Louvet 2007). AH is probably seen primarily in patients with a pre-existing chronic alcoholic liver disease, most often alcoholic cirrhosis, but also occurs in people who have been healthy so far. AH can thus occur both as acute-on-chronic liver failure and as acute liver failure.

**Bacterial translocation as a pathogenetic factor**

There is increasing evidence that bacterial translocation is an early and pathogenetically important event in the development of acute-on-chronic liver failure, ie. suddenly worsening of liver function in patients who already have a degree of liver disease, regardless of the cause of this. Our hypothesis is that this mechanism may also be important in the development of alcoholic hepatitis.

In bacterial translocation, bacteria and bacterial products pass to the lymph and blood vessels. Several conditions with prolonged considerable alcohol intake and with alcoholic liver disease can lead to increased bacterial translocation. Prolonged alcohol exposure to the intestinal mucosa thus leads to the destruction of the *tight junctions*of the epithelium with increased intestinal permeability resulting in increased endotoxin level in the blood^1-3^. Furthermore, the amount of lipopolysaccharide (LPS) producing bacteria in the small intestine (bacterial overgrowth) is increased in cirrhosis, probably as a consequence of a prolonged, large alcohol intake. The combination of cirrhosis and high alcohol consumption thus increases the risk of bacterial translocation ^4; 5^.

**Bacterial translocation and hepatic and systemic inflammation**

Most frequently bacterial translocation is studied by the measurement of bacterial endotoxin in the blood (typically by measuring the LPS from the cell walls of Gram-negative bacteria). The LPS level in blood is elevated in cirrhosis^6^and decreased in treatment with rifaximin ^7^.

As stated, it is believed that bacterial translocation may be important for decompensation of a pre-existing liver disease. Thus, spontaneous bacterial peritonitis (SBP) seen in decompensated liver disease including alcoholic hepatitis is likely a consequence of translocation ^8^. There is also evidence that endotoxin levels in the blood are crucial for the degree of hepatic and systemic inflammation, and alcoholic liver disease is often accompanied by chronic inflammation ^9^.

Endotoxin initiates an inflammatory response by binding to macrophages and monocytes in particular ^10^. The liver houses the body's largest pool of macrophages which are activated by endotoxin to release pro-inflammatory (TNF-alpha, IL1, -2 and IL-6, -8) but also anti-inflammatory cytokines (IL-10). In healthy people, endotoxin is rapidly neutralized in the liver, but alcohol increases the inflammatory response and reduces the liver's ability to clear endotoxin ^11; 12^, which can be further shunted by cirrhosis in the liver. The spleen contains a large pool of monocytes which are also activated by endotoxin to release pro-inflammatory cytokines.

It is possible that the inflammatory state in alcoholic hepatitis occurs as a result of the proinflammatory effects of alcohol and bacterial translocation. This is partly due to direct effects of prolonged alcohol intake (increased intestinal permeability, influence of proinflammatory markers e.g. NF-κB and formation of free oxygen radicals ^13^), partly via a weakened liver function and influence of the liver macrophage system which forms the background for an uncontrolled inflammatory *response*to bacterial translocation (endotoxin effect) ^14^. The inflammatory response in alcoholic hepatitis is massive and is believed to play a crucial role in the development of the often fatal multiorgan failure ^15^, where in addition to liver failure with portal hypertension, variceal bleeding and ascites formation  varying degrees of hepatic encephalopathy and renal failure is seen.

**Rifaximin**

Rifaximin is a non-absorbable broad-spectrum antibiotic which in controlled studies isshown to protect against reoccurrende of hepatic encephalopathy (HE) in cirrhotic patients with previous HE. The preventive effect has now been documented for up to 24 months ^15; 16; 17^.

It is also shown that rifaximin reduces portal pressure and possibly prolongs survival of patients who respond with a reduction of portal pressure by rifaximin treatment^18^. In addition, effects on other cirrhosis-related complications such as variceal bleeding, SBP and HRS^19^ have been described .

It is believed that these systemic and clinical effects of rifaximin are exerted via an effect on the microbiome, ie. the intestinal flora and its metabolic products. Apparently, the effect of rifaximin is primarily a modulation of the bacteria's behavior in the direction of, among other things, reduced toxin production. Accordingly, level of circulating endotoxin and cytokines in cirrhosis is reduced  during rifaximin treatment ^20^.

The optimal dose of rifaximin is not known. The preparation has been used for travel diarrhea for a number of years. Used for this indication, the dose is 200 mg every 8 hours. In studies involving irritable bowel syndrome, daily doses up to 1200 mg have been used. In the treatment of hepatic encephalopathy in patients with cirrhosis, the recommended dose is 550 mg twice daily; this is based on few studies in which this dose was chosen empirically. Cirrhosis patients have a chronic low-inflammatory condition. The inflammation in alcoholic hepatitis is pronounced and self-aggravating, and patients often have intermittent difficulties with tablet intake, which is why dosing divided into three doses throughout the day seems appropriate to ensure a minimum intake of the currently used dose in cirrhosis. Since the antibiotic is not absorbed, the favorable modulation of the domestic flora is likely to become more constant and effective.

**Hepatic encephalopathy**

The mechanism behind the preventive effect on the development of hepatic encephalopathy is not known, but HE is traditionally described as a result of a defect *intestinal liver-brain axis*, wherein the potentially toxic substances formed in the intestine, are not sufficiently detoxified because of impaired liver function. A substance that can experimentally and to some extent also clinically be a candidate as such a toxin is ammonium^21^. Other theories are based on the consistent finding that the blood's content of branched-chain amino acids relative to aromatic amino acids is reduced by cirrhosis ^22^.

**The experiment**

We wish to investigate inflammatory, metabolic and hemodynamic changes in patients with alcoholic hepatitis before, during and after treatment with rifaximin.

**Main purpose**

To investigate the effect of rifaximin on inflammatory and metabolic mediators in patients with alcoholic hepatitis.

Secondarily, to investigate a possible effect on liver function, portal pressure, disturbances of consciousness due to hepatic impairment (hepatic encephalopathy) and renal function.

**Endpoints**

Primary:

Change in blood concentration during rifaximin treatment of:

1. Endotoxin expressed by LPS
2. Inflammation markers: cytokines (IL-1,2,6,8,10, TNF-α), CD163, procalcitonine
3. Metabolic products: glutamine, leucine, isoleucine, valine, phenylalanine, tyrosine, tryptophane, arterial ammonium concentration.

Secondary:

To monitor complications of alcoholic hepatitis and the effects of rifaximin treatment:

1. Post-systemic pressure gradient measured by hepatic catheterization initially and after 3 months
2. Liver function expressed by galactose elimination capacity (GEC) initially and after 3 months
3. Degree of renal impairment: Creatinine, urea, creatinine clearance
4. Hepatic encephalopathy: continuous reaction test, artery-ammonium, number connection test. Initially and after 3 months.

**Patients and method**

**Design**

The study is an exploratory, unblinded and unpaired group comparison experiment. In which 15 patients are included in each group.

Patients with clinical alcoholic hepatitis are included and randomized to

1. Standard medical therapy (SMT*), or
2. Rifaximin treatment combined with SMT*

*According to Danish guidelines for alcoholic hepatitis SMT includes treatment with pentoxifylline 400 mg x 3 daily for 2-4 weeks. In addition, patients are given nutrition, fluid therapy, systemic antibiotics and possibly Vitamin K.

**Experimental medicine:**

Rifaximin (Xifaxan) 550 mg tablets will be given by the nurse to the patient for oral administration at 8-hour intervals for 4 weeks. The nurse observes that the tablet is being taken. If the patient's level of consciousness does not allow oral ingestion, the tablets will be administered via a gastric tube, which must be frequently used in this patient group and which is used for other medication and nutrition. Only patients who take at least 80% of the project medicine will be included in the data material. The medicine is used from regular stock.

Each patient who receives rifaximin treatment receive a personal labeled pill bottle (including the addition of patient ID number and name of investigator), which is stored in a locked medicine depot. The additional labelling takes place in as specified in GMP Annex 13. This will enable counting and provide further assurance that the patient has received the correct number of tablets during the trial period. If the patients are discharged within the four weeks of treatment with rifaximin, the pill bottle can be given along for continued intake at home / at the local hospital. Unused medication / empty pill bottles are returned for documentation of compliance.

**Side effects**

**Adverse reactions according to the catalog of medicines:**It is stated in the catalog of medicines that most of the side effects described are probably not real side effects but are part of the underlying disease being treated. Thus, the gastrointestinal symptoms are frequently present when rifaximin is used to treat travel diarrhea, while fever, ascites, anemia, arthralgia and depression are frequent manifestations in patients treated with rifaximin for hepatic encephalopathy.

Overall, all of the described frequent and non-frequent side effects may occur as part of the underlying disease in  patients with liver cirrhosis and hepatic encephalopathy and patients with infectious gastroenteritis, which are the two conditions with approved indication for treatment with rifaximin, and as a starting point only significant change in intestinal function during treatment is expected to be perceived as a side effect. This is also due to the poor absorption of the drug, a maximum of 7 % is reported in absorption studies to be absorbed; the drug catalog lists a maximum of 1 % absorption.

Common (1-10 %) : Temperature rise, nausea, vomiting, gastrointestinal upset, abdominal pain , diarrhea, constipation, flatulence , ascites*, peripheral edema, anemia*, arthralgia*, muscle cramps, headache, dizziness, depression*, skin rash, itchy skin.

Uncommon (0.1 % -1 %): Flu-like symptoms, Hematochezia, hypertension, palpitations, dyspnea, lymphocytosis, neutropenia, myalgia, abnormal dreams, insomnia, somnolence, migraine, paresthesia, candidiasis, hematuria, double vision.

Rare (0.01-0.1 %): Hypotension*, pneumonia*.

Unknown frequency: Thrombocytopenia, syncope, anaphylactic reaction, angioedema.

****Only seen in treatment aiming to reduce recurrence of manifest hepatic encephalopathy.***

**Side effects described in the Summary of Product Characteristics for rifaximin**

This describes side effects from clinical studies, in which 322 patients were treated with rifaximin 550 mg twice daily for 12 months (66 % of patients) and for 24 months (39% of patients), and 152 patients with hepatic encephalopathy were treated in three supportive studies with varying doses of rifaximin from 600 mg to 2400 mg per day for up to 14 days.

All adverse events that occurred in patients treated with rifaximin with an incidence ≥5 % and a higher incidence (≥1 %) than placebo patients in the former study are listed in the following table.

| **MEDDRA**  **System organ class** | **Incident** | **Placebo**  **N = 159**  **n %** | | **Rifaximin**  **N = 140**  **n %** | |
| --- | --- | --- | --- | --- | --- |
| **Blood and lymphatic system** | Anemia | 6 | 3.8 | 11 | 7.9 |
| **Gastrointestinal tract** | Ascites | 15 | 9.4 | 16 | 11.4 |
|  | Nausea | 21 | 13.2 | 20 | 14.3 |
|  | Pain in the upper abdominal cavity | 8 | 5.0 | 9 | 6.4 |
| **General disorders and administration site conditions** | Peripheral edema | 13 | 8.2 | 21 | 15.0 |
|  | Pyrexia | 5 | 3.1 | 9 | 6.4 |
| **Bones, joints, muscles and connective tissue** | Muscle spasms | 11 | 6.9 | 13 | 9.3 |
|  | Arthralgia | 4 | 2.5 | 9 | 6.4 |
| **The nervous system** | Dizziness | 13 | 8.2 | 18 | 12.9 |
| **Mental disorders** | Depression | 8 | 5.0 | 10 | 7.1 |
| **Respiratory, thoracic and mediastinal disorders** | Dyspnoea | 7 | 4.4 | 9 | 6.4 |
| **Skin and subcutaneous tissue** | Itch | 10 | 6.3 | 13 | 9.3 |
|  | Rash | 6 | 3.8 | 7 | 5.0 |

**Registration of events / potential side effects:**

All new symptoms and events will be registered on a daily basis by medical supervision and registered in the subject's CRF. At the same time, it is assessed whether the symptom / event can be attributed to the underlying disease or must be perceived as a potential side effect of rifaximin treatment. For this, the summary of product characteristics is used as a reference document. If a side effect is suspected, its severity is graded and, prior to the next planned medication administration, a decision is made as to whether the treatment should be discontinued.

Patients who have adverse events / side effects will be monitored appropriately and clinical assessments, laboratory tests and treatments will be initiated at the discretion of the treating physician. All adverse events / side effects will be followed up for satisfactory recovery or stabilization.

Investigator immediately reports all SAEs (see definition below) to the sponsor. Sponsor evaluate all SAE and SAR and ensure that all information about unexpected serious suspected adverse reactions (SUSARs) that are fatal or life-threatening is recorded and reported to the National Board of Health and the Research Ethics Committee as soon as possible and no later than 7 days after the sponsor has knowledge such a presumed side effect. Any SUSAR will be reported via the Danish Health and Medicines Authority's e-form.

No later than 8 days after the report, the sponsor informs the National Board of Health of all relevant information about the sponsors and investigators follow-up on the report.

All other unexpected and serious suspected side effects must be reported to the National Board of Health no later than 15 days after the sponsor has become aware of these.

Symptoms expected from the underlying disease (alcoholic hepatitis) or complications thereof (including hepatic encephalopathy, ascites, variceal bleeding, renal failure, nausea / vomiting), as well as common symptoms from any other known diseases in the subject will not be recorded.

Biochemical events are defined as clinically significant deviations in the subjects' blood samples during the trial, which give rise to suspicion of disease or organ damage not due to the underlying disease or its complications, and of such a degree that it requires active treatment. Thus, elevated creatinine, urea, bilirubin, leukocyte count, INR, CRP and ammonium, as well as decreased hemoglobin, platelet count, PP and albumin are most commonly seen in patients with alcoholic hepatitis, so finding these will not lead to reporting unless the deviation is of an order that is unusual in alcoholic hepatitis.

In the trial, adverse events and side effects are defined as follows:

- *Adverse event (Adverse Event, AE):*Any adverse event in a patient following treatment with the test drug, without necessarily correlation between the test drug and the adverse event.
- *Adverse Reaction (AR):*Any harmful and unwanted reaction to a trial *drug,*regardless of dose (AR) (= causality between the trial drug and the adverse event).
- *Serious incident ( Serious Adverse Event SAE) or serious suspected adverse reaction ( Serious Adverse Reaction, SAR):*An adverse event or suspected adverse reaction that at any dose results in death, is life-threatening, requires hospitalization or prolongation of existing hospitalization, results in persistent or significant disability or incapacity for work or leads to a congenital anomaly or malformation.
- *Unexpected side effect (Unexpected Adverse Reaction):*A suspected adverse reaction, the nature or severity is not consistent with the product information. If this is also severe it is called: *Suspected Unexpected Serious Adverse Reaction, SUSAR.*

The Summary of Product Characteristics for rifaximin is used as a reference document when assessing whether an adverse reaction is unexpected or expected.

AE / AR is registered in CRF during the 4 weeks where the medicine is taken. Patients are openly asked if there have been any new symptoms since the last visit. The association between the adverse event and the experimental drug is recorded for each adverse event.

A list of SARs that have occurred during the trial period and a report on the safety of the subjects are submitted once a year to the National Board of Health and to the local Science Ethics Committee.

A list of all AEs and ARs will appear in the final report, which will be submitted no later than 90 days after the end of the trial to the National Board of Health and to the local Science Ethics Committee.

**Subjects**

All patients hospitalized for alcoholic hepatitis in Hepatology clinic A, Rigshospitalet, from 1^st^ of june 2014 to 31^st^ of  May 2017 are eligible for the study, and will be asked to participate. If the patient has a degree of HE, the patient's next of kin and own doctor will be consulted regarding deputy consent.

**Inclusion criteria**

- Alcohol consumption; more than 3 units daily for more than 3 months, or more than 10 units daily for at least one month
- Rapid development of jaundice over 14 days with bilirubin above 50 micromol / l
- Imaging and exclusion of mechanical / obstructive jaundice

**Exclusion criteria**

- Pregnancy*
- Patients under 18 years of age
- Persons unable to give informed consent
- Patients who have been treated with rifaximin within the past month
- Malignancy
- Allergy to any of the ingredients of study medication
- Intestinal obstruction (contraindicates rifaximin treatment)

*Pregnancy tests ensure that fertile women are not pregnant at the time of inclusion. During the trial period, the patients are hospitalized and too ill to become pregnant. If a pregnant woman unexpectedly should be discharged before the end of the trial period, she will be thoroughly informed not to become pregnant and use safe contraception during the trial period, ie. IUD or hormonal contraception (birth control pills, prolonged-release injection, implant, transdermal transdermal patch or vaginal ring).

The individual patient will be included in the trial with a total of 4 weeks of medication or SMT. Three months after baseline investigations of the patients will be carried out including liver-vein catheterization with measurement of hepato-venous pressure gradient, elastography, GEC, CRT, track test and blood tests. The three-month follow-up is performed at the department.

**Exclusion from and interruption of trials**

If any serious unexpected adverse reactions are observed the trial will be adjourned and trial participants, National Board of Health and the ethical committees will be notified.

Exclusion from the study ahead of time for the individual trial participant occurs if

- The trial participant withdraws consent
- If the trial participant develops a serious side effect
- If the trial participant does not follow the instructions for participation in the study - eg does not take the trial medicine. The decision on this is made by the person responsible for the experiment.

**Subjects who leave / end the experiment:**

When the subjects finish the experiment, they move on to regular follow-up/ treatment. This most often involves relocation to a local hospital (normal procedure) or discharge. All patients provided follow-up three months later with further characterization of liver function, including liver-vein catheterization, elastography, possible CT scan of the liver, as well as blood tests, support in further alcohol abstinence and treatment of any complications.

Subjects who choose to withdraw from the trial before the planned termination are treated according to the usual regimen (SMT) and are offered the same post-treatment.

If the subject is excluded from the study within the first 7 days of the trial period, no data is registered. If the subject is excluded 7-27 days into the experiment, the data obtained will be retained in CRF, and included in sub-analyzes. For subjects who do not participate throughout the trial period, a “replacement subject” is included so that the data processing is based on complete data from 30 subjects.

**Randomization**

The patients are randomized randomly by drawing lots (envelope method) according to a randomization list that ensures equal distribution of the patients in the two groups assessed on the basis of disease severity assessed by GAHS in the two groups. The randomization is performed by a third person who does not work in the clinic and has no knowledge of the patients.

**Pre-randomization study - up to day 0**

1. Prognostic scoring using scores developed for alcoholic hepatitis / liver disease (GAHS, Maddrey, ABIC and MELD scores), as well as SIRS scoring.
2. Ultrasound or other imaging of the liver to exclude mechanical biliary obstruction
3. Transjugular liver biopsy if possible, otherwise as early in the trial as possible (usual clinical practice).
4. Elastography before day 7.
5. Liver-vein catheterization before day 7.
6. Galactose elimination capacity.

**Intervention**

Treatment with rifaximin is started on day 1 with oral ingestion of rifaximin tablets 550 mg 3 times daily.

Other treatment is adjusted based on usual clinical practice, including the decision on prednisolone treatment, as the Danish guidelines ( http://mit.dsgh.net/images/stories/dsgh/guidelinefiles/alkoholisk%20hepatitis%20gudeline%20newfinalrevision_170513.pdf ) are followed, ie. primarily choice of treatment with pentoxifylline (PTX) in the first 14 days of the disease. In case of clinical improvement, ie. 50 % reduction of the bilirubin level, PTX is continued for another 2 weeks s for a total treatment duration of 4 weeks. In case of insufficient treatment effect of PTX, steroid treatment should be considered. If initiated, the treatment effect is assessed by calculating Lille score^23^ after 7 days of treatment. For Lille scores below 0.45, treatment is continued for a total of 4 weeks. This also applies if patients have started steroid treatment before admission to Hepatology Clinic A.

**Measurements**

Day 0, 7, 14, 21 and 28 about 40 ml of blood are drawn for analysis of the following parameters:

**Inflammation**

1. Endotoxin (LPS)
2. Cytokines (IL-1,2,6,8,10, TNF-alpha )
3. CD 163 (macrophage activation marker)
4. Procalcitonin
5. Blood for biobank for later genetic examination

**Metabolism and HE**

1. Glutamine, isoleucine, leucine, valine, tyrosine, tryptophan and phenylalanine
2. Arterial - ammonium
3. Continuous Response Time Test (CRT)
4. Number connection test

**Renal function**

1. Collection of diurnal urine to determine creatinine clearance

**Examinations day 90**

1. Elastograhpy
2. Liver-vein catheterization
3. Galactose elimination capacity.
4. CRT, number connection test
5. Blood tests and daily urine as at day 0,7,14,21 and 28.

**Procedure descriptions and analysis methods:**

**Blood tests:**

Amino acids (AA) from the plasma are analyzed using Mass Trak ^TM^Amino Acid Solution Kit ^TM 24^. Immediately after sampling, plasma is isolated by centrifugation of the blood and the sample is frozen at -80 ^o^C to avoid decay of the individual AAs, eg Glutamine to Glutamic acid etc. Plasma is treated with the addition of sulfosalicylic acid and 6-aminoquinolyl-N-hydroxysuccinimidyl carbamate and analyzed then via UV- Ultra High Performance chromatography.

Blood for **ammonium**determination is taken via arterial puncture or drawn directly from the arterial cannula. The complications are few and are part of daily practice ^25^. A minimum of 2 ml of blood is put on ice immediately after sampling and cool centrifugation (10 min., 2000g) no later than 30 minutes after sampling is performed, after which the sample is analyzed^26^. The patient should not have smoked 6 hours before sampling.

**Inflammation markers**(IL-1, 2, 6, 8, 10, TNF-alpha and CD-163) are measured in EDTA plasma. Two 9 ml EDTA glasses are sampled. After sampling, the blood is centrifuged, and plasma is pipetted in appropriate micropipettes. For storage, samples are frozen at -80 ^°^C before analysis in a commercial ELISA kit. An additional 4 ml dry glass is sampled for serum cytokine analysis.

Procalcitonine is measured in serum by immunofluorometric assay (BRAHMS Cryptor) ^27^. The sample is routinely analyzed together with other standard blood samples, including C-reactive protein, liver counts, etc.

Blood for use for future analysis of inflammation markers and genetic testing is stored in a dedicated Biobank (-80 ^o^C freezer). The patient consent includes acceptance for storage of tissue material.

Genetic testing is based on RNA. The sample is collected in a commercial 2.5 ml PaxGene Blood RNA glass containing an RNA stabilizer reagent ^28^ and stored for up to 5 years. Blood for future inflammatory marker examination is collected in 9 ml EDTA tubes. Plasma is separated by centrifugation. The sample is also stored at -80 ^o^C for up to 5 years.

**Endotoxin (LPS)**

Endotoxin is measured on 50 microliters of serum with sandwich ELISA test. Serum is pipetted and stored at -80 ^o^C until analysis time. All samples will be analyzed at the same time after collection.

**Continuous Response**Time Test **(CRT)**is a psychometric test to examine brain function and assess early onset consciousness disorder (hepatic encephalopathy) ^29^. The test is computer-based and is performed with Bitmatic EKHO version 1.3.2410.42508 software. Test time is 10 minutes. The test measures the response time between an audio signal emitted from the computer and until the patient has detected the sound and pressed a button.

**Number connection test**is as CRT a psychometric test for the assessment of disturbances of consciousness in liver patients with possible hepatic encephalopathy. The patient is given a piece of pre-printed paper with a randomly distributed number sequence (type A) or a combination of numbers and letters (type B). The time is measured from start to correct completion, ie correct chronological connection of numbers / numbers + letters, including any correction time. Test type A is connected 1-2-3-4-etc. Type B 1-A-2-B-3-C-etc.

**Galactose elimination capacity (GEC)**is a quantitative study to determine the metabolic capacity of the liver ^30; 31^. The study is performed by injecting a galactose solution intravenously over 5 minutes (1 ml / kg body weight of 500 mg / ml galactose solution). Capillary blood is drawn every 5 minutes for the period 20-45 minutes after the injection and the galactose blood concentration is determined. In parallel, urine is collected for 4 hours to determine the urinary excretion of galactose. Via formula, a number is calculated for GEC. The study is not associated with discomfort or risks to the patient.

**Hepatic catheterization (LVK)**is performed for measurement of the pressure difference in the portal circuit across the liver, ie. a determination of the pressure gradient between the portal vein (the blood vessel from the intestine to the liver) and the caval vein (the large blood vessel returning blood from the body, including the liver, to the heart). The pressure gradient reflects the severity of connective tissue formation in the liver.

The patient is fasting but can take regular medication except diuretics and insulin. The procedure has a duration of approximately 45 minutes. The patient is placed in a supine position and is fully conscious. After the procedure, there are no restrictions. The procedure is otherwise part of the standard patient assessment ^32^.

The procedure is performed sterile and with the insertion of a peripheral venous catheter (PVK) in the right elbow bend. A guide wire is inserted and after local anesthesia and a 2 mm short cut in the skin replaced with a short 2 mm thick insertion sleeve. This introduces the hepatic venous catheter itself, which is positioned in the hepatic vein using imaging for guidance.

The catheter is connected to a calibrated pressure transducer and a number of pressure measurements are performed in addition to measuring pressure in the vena cava above and below the liver, and possibly in right atrium and ventricle. Similar procedure for similar patient material described as safe and with few risks ^33^.

**Transjugular liver biopsy is**performed rather than percutaneous biopsy due to the patient group's often significant coagulopathy and frequent occurrence of ascites. The procedure is part of routine clinical assessment^34^. The biopsy is secured through catheter-based technique first with ultrasound-guided access to the internal jugular vein (IJV) and finally biopsy with imaging guidance. Discomfort during the examination is rare and in general the procedure is risk-free ^35^.

Local anesthesia is applied at the injection site on the neck, typically on the right side, and sterile conditions are ensured. First a needle is inserted ultrasound-guided and this is replaced with an insertion sleeve. A catheter is then passed via the insertion sheath through the IJV to a suitable hepatic vein, where the biopsy is obtained. During biopsy, imaging is used to ensure correct placement in the hepatic vein in relation to the liver tissue.

The patient fasts prior to the examination; including medicine. The procedure is performed with the patient fully awake and lying on an examination table. After biopsy, the patient is observed in the ward for 2 hours for changes in blood pressure, heart rate and abdominal pain, just as the patient is monitored with ECG electrodes and pulse oximetry ^36^during the procedure.

**Ultrasound scan (US)** of the abdomen is performed at time of diagnosis to rule out mechanical / obstructive jaundice. This follows common standards and is not associated with any risk for the patient.

**Fibroscan**or *transient elastography*measures the elasticity of the liver. The procedure is not associated with risks or inconveniences to the patient and can be compared to conventional US scan. The transducer has a built-in pulse generator that sends low-frequency pressure waves through the liver.  US-measured propagation speed is converted to a number characterizing liver stiffness. The measurement is repeated until there are ten technically satisfactory measurements. The examination is performed with the patient in supine position and with the right arm under the head in maximum abduction. The procedure is preceded by 2 hours of fasting^37^.

Data collection

For each patient, a CRF is prepared which is marked with the patient's project ID number. It records the randomization group and the nature of standard treatment (pentoxifylline / prednisolone), the patient's age and sex, symptoms of hospitalization, alcohol history, blood test results, ultrasound examination, hepatic venous catheterization, elastography, creatinine clearance and liver biopsy. In addition, any infections, antibiotic treatment, implantation of bladder catheter, implantation of gastric tube and any complications such as side effects of treatment, renal failure, bleeding from varicose veins, ascites or hepatic encephalopathy and resultant examinations / treatment in connection with these are registered.

All the above data are already registered electronically in the patient's medical record and digital imaging, laboratory and pathology systems, although the alcohol history is sought to be even more precise than usual. All new symptoms are registered on a daily basis, in addition, whether the symptom is considered to be a possible side effect of rifaximin treatment is registered.

Investigator allows direct access to source data / documents (including patient records) by monitoring, auditing and / or inspection from the GCP units and the National Board of Health.

**Statistics**

Differences are examined for statistical significance in ANOVA. A P-value below 0.05 will be considered statistically significant.

Data from 30 evaluable subjects will be included in the statistical analyses. Strength calculation (www.stat.ubc.ca) has been performed based on data regarding endotoxin (LPS) level after rifaximin treatment, as this is expected to be halved.

In addition, subjects who have participated in a minimum of 7 days of the experiment will be included in sub-analyses.

**Information for participants**

All subjects receive both written information and thorough oral information about the study. First participants will receive oral information about the project from one of the physicians involved in the project. If the patient is interested in participating, written participant information will be provided. A new appointment for further oral information from one of the project affiliated physicians will be arranged and volunteers will be informed that it is possible to bring an assessor to this conversation. Subjects will be informed about the right of consideration up to one day after the information, written consent can, however, also be signed in connection with this conversation. The oral information will take place undisturbed in an enclosed room according to the enclosed guidelines for oral information.

Included and potential test subjects can obtain further information about the project by contacting the doctors responsible for the project: Henriette Ytting, Ole Hamberg, Mette Kjær and Johan Rohde by telephone or e-mail. At request, the written participant information, the trial protocol and the protocol summary can be handed out by the project managers.

**Registration of information and professional secrecy**

The recorded data will be stored for up to 15 years after the end of the experiment and will be used in scientific inventories. All persons associated with the project have a duty of confidentiality and will treat the personal data in strict confidence. At no time will information be passed on about health conditions and other purely private matters that can be attributed to the individual subjects. All information regarding the subjects' identities is protected in accordance with current rules in the Health Act. The law on the processing of personal data will be complied with and the project has been notified to the Danish Data Protection Agency under Rigshospitalet's joint notification.

***Disclosure***

Based on the collected data, the following issues will be highlighted:

1. The effect of rifaximin treatment on inflammatory, metabolic and hemodynamic parameters
2. Tolerance / safety of treatment. No previous studies have found side effects or adverse effects of the treatment.

The results of this study are expected to result in several publications in international scientific journals with peer-review. In addition, the results will be presented at national and international meetings within the area. Both positive, negative and inconclusive results will be published.

The authors of articles will adhere to the Vancouver Declaration and have actively contributed to the project.

Reporting to authorities

No later than 90 days after the last subject's last project visit, the authorities will be notified that the trial has been completed. Thereafter, a final clinical report for the trial will be submitted within 12 months.

***Economic conditions***

The project is initiated by the chief physician, Ph.D. Henriette Ytting and head of clinic, Med.Sc.D Ole Hamberg.

The study is funded by the participating departments. Funds will be applied for to complete the project, including remuneration of Ph.D. students. The National Board of Health and the Science Ethics Committee will be informed if financial support is obtained.

None of the project participants have financial interests in the project.

**Biological material**

The urine sample and some of the blood samples will be analyzed immediately, ie within a maximum of 7 days. Other blood samples will be stored in a research biobank and analyzed by the end of the project. Any excess blood will subsequently be transferred to a biobank. The blood will not be taken out of the country, it will be used for analyses regarding markers for alcoholic hepatitis if the need arises. This blood will only be tested after renewed permission from a scientific ethics committee and the blood will be stored in the biobank with permission from the Data Inspectorate.

The trial participants can request destruction of the biological material that has not yet been analysed if they so wish at a later date.

**Usefulness of the trial**

The individual subject may not benefit from the project. It is expected that the trial can contribute useful knowledge that will benefit future patients. It is possible that the trial participants who receive rifaximin benefit from this.

**Side effects, risks and disadvantages**

There are no extra inserts for blood sampling, but a total of 240 ml of extra blood is drawn for the project. Side effects can occur with treatment with rifaximin (please see the section on this) but it is stated in the profile of the side effect of the preparation that the listed side effects are probably associated with the disease being treated and not with rifaximin. Liver biopsy, GEC measurement, diurnal urine collection, liver vein catheterization, elastography, reaction time measurement and diagnostic imaging is performed as part of the general diagnostics and treatment.

**Ethical considerations**

All subjects sign a declaration of consent informing them of their option to withdraw from the project at any time, without this having any bearing on their future treatment.

As described above, participation in the trial is not considered to expose the patient to any additional risks. Based on existing knowledge, an immediate benefit is expected for those of the participating patients receiving rifaximin treatment. The subjects are offered to be informed of the results of their own examinations and blood tests by the end of the project, but there are no other immediate benefits for the individual by participating in the trial. Furthermore, additional basal knowledge about changes in metabolism, inflammatory response and complications during rifaximin treatment will be obtained.

It is therefore considered ethically justifiable to carry out this project in this group of seriously ill patients.

**Practical conditions**

The patients will be treated at the Department of Hepatology, Rigshospitalet. The department has a highly specialized function for treatment of patients with acute and acute-on-chronic liver failure, which this trial deals with, and it is assessed that among the people associated with the project the necessary expertise to carry out the project is available.

The project is expected to be completed in September 2017.

**Reference List**

              (1) Bjarnason I, Peters TJ, Wise RJ. The leaky gut of alcoholism: possible route of entry for toxic compounds. *Lancet*1984; **1**(8370): 179-82.

              (2) Fukui H, Brauner B, Bode JC *et al*. Plasma endotoxin concentrations in patients with alcoholic and non-alcoholic liver disease: reevaluation with an improved chromogenic assay. *J Hepatol*1991; **12**(2): 162-9.

              (3) Keshavarzian A, Farhadi A, Forsyth CB *et al*. Evidence that chronic alcohol exposure promotes intestinal oxidative stress, intestinal hyperpermeability and endotoxemia prior to development of alcoholic steatohepatitis in rats. *J Hepatol*2009; **50**(3): 538-47.

              (4) BauerTM, Steinbruckner B, Brinkmann FE *et al*. Small intestinal bacterial overgrowth in patients with cirrhosis: prevalence and relation with spontaneous bacterial peritonitis. *Am J Gastroenterol*2001; **96**(10): 2962-7.

              (5) Hauge T, Persson J, Danielsson D. Mucosal bacterial growth in the upper gastrointestinal tract in alcoholics (heavy drinkers). *Digestion*1997; **58**(6): 591-5.

              (6) Albillos A, de la HA, Gonzalez M *et al*. Increased lipopolysaccharide binding protein in cirrhotic patients with marked immune and hemodynamic derangement. *Hepatology*2003; **37**(1): 208-17.

              (7) Vlachogiannakos J. Intestinal decontamination improves liver haemodynamics in patients with alcohol-related decompensated cirrhosis. 2009.

              (8) Wiest R, Lawson M, Geuking M. Pathological bacterial translocation in liver cirrhosis. *J Hepatol*2014; **60**(1): 197-209.

              (9) McClain CJ, Shedlofsky S, Barve S *et al*. Cytokines and alcoholic liver disease. *Alcohol Health Res World*1997; **21**(4): 317-20.

              (10) Heeg K, Sparwasser T, Lipford GB *et al*. Bacterial DNA as an evolutionary conserved ligand signaling danger of infection to immune cells. *Eur J Clin Microbiol Infect Dis*1998; **17**(7): 464-9.

              (11) Fukui H, Kitano H, Okamoto Y *et al*. Interaction of Kupffer cells to splenic macrophages and hepatocytes in endotoxin clearance: effect of alcohol. *J Gastroenterol Hepatol*1995; **Suppl 1**: S31-S34.

              (12) McClain CJ, Barve S, Barve S *et al*. Tumor necrosis factor and alcoholic liver disease. *Alcohol Clin Exp Res*1998; **22**(5 Suppl): 248S-52S.

              (13) Gloire G, Legrand-Poels S, Piette J. NF-kappaB activation by reactive oxygen species: fifteen years later. *Biochem Pharmacol*2006; **72**(11): 1493-505.

              (14) Lin RS, Lee FY, Lee SD *et al*. Endotoxemia in patients with chronic liver disease: relationship to severity of liver disease, presence of esophageal varices, and hyperdynamic circulation. *J Hepatol*1995; **22**(2): 165-72.

              (15) Fukui H. Relation of endotoxin, endotoxin binding proteins and macrophages to severe alcoholic liver injury and multiple organ failure. *Alcohol Clin Exp Res*2005; **29**(11 Suppl): 172S-9S.

              (16) Bass NM, Mullen KD, Sanyal A *et al*. Rifaximin treatment in hepatic encephalopathy. *N Engl J Med*2010; **362**(12): 1071-81.

              (17) Mullen KD, Sanyal AJ, Bass NM *et al*. Rifaximin Is Safe and Well Tolerated for Long-term Maintenance of Remission From Overt Hepatic Encephalopathy. *Clin Gastroenterol Hepatol*2013.

              (18) Vlachogiannakos J. Intestinal decontamination improves liver haemodynamics in patients with alcohol-related decompensated cirrhosis. 2009.

              (19) Vlachogiannakos J, Viazis N, Vasianopoulou P *et al*. Long-term administration of rifaximin improves the prognosis of patients with decompensated alcoholic cirrhosis. *J Gastroenterol Hepatol*2013; **28**(3): 450-5.

              (20) Kalambokis GN, Mouzaki A, Rodi M *et al*. Rifaximin improves systemic hemodynamics and renal function in patients with alcohol-related cirrhosis and ascites. *Clin Gastroenterol Hepatol*2012; **10**(7): 815-8.

              (21) VoorhiesTM, Ehrlich ME, Duffy TE *et al*. Acute hyperammonemia in the young primate: physiologic and neuropathologic correlates. *Pediatr Res*1983; **17**(12): 970-5.

              (22) Eriksson LS, Conn HO. Branched-chain amino acids in the management of hepatic encephalopathy: an analysis of variants. *Hepatology*1989; **10**(2): 228-46.

              (23) Louvet A, Naveau S, Abdelnour M *et al*. The Lille model: a new tool for therapeutic strategy in patients with severe alcoholic hepatitis treated with steroids. *Hepatology*2007; **45**(6): 1348-54.

              (24) Hong, P. Analysis of Physiological amino acids with the Mass Trak ^TM^amino acid analysis solution . 2009. Ref Type: Generic

              (25) Slogoff S. On the safety of radial artery cannulation. 1983.

              (26) Rigshospitalet. Rigshospitalet's laboratory guidance. 2014. Ref Type: Generic

              (27) Rigshospitalet. Rigshospitalet's laboratory guidance. 2014. Ref Type: Generic

              (28) Moller HJ. Characterization of an enzyme-linked immunosorbent assay for soluble CD163. 2002.

              (29) PreAnalytix. PreAnalytix. 2014. Ref Type: Generic

              (30) Lauridsen MM. Critical flicker frequency and continuous reaction times for the diagnosis of minimal hepatic encephalopathy: a comparative study of 154 patients with liver disease. 2011.

              (31) Tygstrup N. Determination of the hepatic elimination capacity (Lm) of galactose by single injection. 1966

              (32) Tygstrup N. Effect of sites of blood sampling in determination of the galactose elimination capacity. 1977.

              (33) Clausen AR. Hepatic venous catheterization (LVK). 2012. Ref Type: Generic

              (34) Jacobs E. Safety of transradial cardiac catheterization in patients with end-stage liver disease. 2014.

              (35) Kalambokis G. Transjugular liver biopsy - indications, adequacy, quality of specimens, and complications - a systematic review. 2007.

              (36) Radiology Clinic X. Transjugular liver biopsy - 2014. Ref Type: Generic

              (37) Mossner BK. [Transient elastography for diagnosing liver fibrosis]. 2008.
